# Tumor Immune Microenvironment Characterization in Hepatocellular Carcinoma Identifies Four Prognostic and Immunotherapeutically Relevant Subclasses

**DOI:** 10.3389/fonc.2020.610513

**Published:** 2021-02-19

**Authors:** Xingxing Gao, Hechen Huang, Yubo Wang, Caixu Pan, Shengyong Yin, Lin Zhou, Shusen Zheng

**Affiliations:** ^1^Division of Hepatobiliary and Pancreatic Surgery, Department of Surgery, The First Affiliated Hospital, Zhejiang University School of Medicine, Hangzhou, China; ^2^NHC Key Laboratory of Combined Multi-organ Transplantation, Hangzhou, China; ^3^Key Laboratory of Organ Transplantation, Research Center for Diagnosis and Treatment of Hepatobiliary Diseases, Hangzhou, China

**Keywords:** tumor microenvironment, carcinoma, hepatocellular, cancer immunology, classification, machine learning

## Abstract

**Purpose:**

The tumor microenvironment (TME) plays a critical role in the pathogenesis of hepatocellular carcinoma (HCC). However, underlying compositions and functions that drive the establishment and maintenance of the TME classifications are less-well understood.

**Methods:**

A total of 766 HCC patients from three public cohorts were clustered into four immune-related subclasses based on 13 TME signatures (11 immune-related cells and 2 immune-related pathways) calculated by MCP-counter. After analyzing the landscapes of functional annotation, methylation, somatic mutation, and clinical characteristics, we built a TME-based Support Vector Machine of 365 patients (discovery phase) and 401 patients (validation phase). We applied this SVM model on another two independent cohorts of patients who received sorafenib/pembrolizumab treatment.

**Results:**

About 33% of patients displayed an immune desert pattern. The other subclasses were different in abundance of tumor infiltrating cells. The Immunogenic subclass (17%) associated with the best prognosis presented a massive T cell infiltration and an activation of immune checkpoint pathway. The 13 TME signatures showed a good potential to predict the TME classification (average AUC = 88%). Molecular characteristics of immunohistochemistry from Zhejiang cohort supported our SVM classification. The optimum response to pembrolizumab (78%) and sorafenib (81%) was observed in patients belonging to the Immunogenic subclass.

**Conclusions:**

The HCC patients from distinct immune subclass showed significant differences in clinical prognosis and response to personalized treatment. Based on tumor transcriptome data, our workflow can help to predict the clinical outcomes and to find appropriate treatment strategies for HCC patients.

## Introduction

Hepatocellular carcinoma (HCC), the predominant type of primary liver cancer, is the fourth leading cause of cancer mortality worldwide with about 782,000 deaths annually (1). HCC is strongly influenced by the tumor microenvironment (TME), as well as reported to benefit from the immune-checkpoint blockade treatment. As an inflammation-related tumor, the specific TME of HCC can influence the immune tolerance and evasion by mixed mechanisms. Numerous studies have been reported that the TME plays a critical role in tumor initiation, progression, and outcome (2, 3). The TME is an intricate system, which coexists and interacts with cancer cells, immune cell subsets, extracellular matrix, various cytokine, and other unknown components to maintain the tumorigenesis of HCC (4). The complexity of the TME relies on the immune infiltration, as tumors could be classified into the tumor immunity continuum (5). Hegde et al. suggested that human tumors could be categorized as inflamed, immune desert, or immune-excluded phenotypes correspond to different mechanisms of immune response and escape (6). Thorsson et al. proposed a classification of six immune subclasses (wound healing, lymphocyte depleted, TGF-β dominant, inflammatory, immunologically quiet, IFN-γ dominant), based on extensive immunogenomic analysis of 33 cancer types compiled by TCGA (7).

Meanwhile, estimating the cellular composition of the TME requires accurate and robust methods. Fluorescence-activated cell sorting (FACS) operates only a small number of cell type-specific markers and requires large amounts of fresh tumor tissues, which limit the applications on tumor biopsies (8). Single-cell sequencing has a high precision, but currently it is too expensive for large-scale clinical application (9). In order to overcome the above shortcomings, we turn to the methods for high-throughput technologies applied in clinical settings, which TME are inferred using computational algorithms. High throughput technologies, such as RNA-Seq and microarray, provide large-scaled transcriptome data and offer opportunity for estimation of the abundance of tumor infiltrated immune cells. Several methods like Microenvironment Cell Population-counter (MCP-counter), CIBERSORT and TIMER have been developed to robustly and precisely quantify immune cells using transcriptome data obtained from bulk tissue specimens (10–12).

After exploring the distinct compositions and functions of the TME by MCP-counter, a total of 766 HCC patients from three public cohorts were clustered into four subclasses (namely Immune desert, Immunogenic, Innate immune and Mesenchymal) based on 13 TME signatures. Furthermore, a Support Vector Machine (SVM) was constructed to predict the HCC classification (average AUC = 88%). Finally, by applying our SVM model on another two independent cohorts of patients who received sorafenib/pembrolizumab treatment, we found that patients classified into Immunogenic subclass showed the highest response rate to sorafenib (81%) and pembrolizumab (78%). Thus, we suggested that HCC patients may benefit from identifying the immune subclass which infer clinical outcomes and guide personalized treatment strategies (**Supplementary Figure 1**).

## Materials and Methods

### Ethics Statement and Consent for Publication

The studies involving human participants were reviewed and approved by Clinical Research Ethics Committee of the First Affiliated Hospital College of Medicine, Zhejiang University (2014-334). Operation informed consents and Informed consent form for scientific research were obtained from all participants for the publication of any potentially identifiable images or data included in this article.

### Clinical Cohorts and Preprocessing

Three public transcriptome data sets were enrolled in our study, including the TCGA-LIHC cohort of the Cancer Genome Atlas (n = 365), the CHCC-HBV cohort of Gao et al. (n = 159) and the GSE14520 cohort of Roessler et al. (n = 242) (13, 14). Among above data sets, any case with null value of survival information had been excluded. As to TCGA-LIHC and CHCC-HBV cohort, the fragments per kilobase per million (FPKM) data and clinic information were downloaded from the UCSC Xena (xenabrowser.net) and The National Omics Data Encycolpedia (www.biosino.org/node), respectively. The “CEL” files of GSE14520 were downloaded and normalized by the “frma” function using frma (R package). Besides, GSE78220 (28 melanoma patients received pembrolizumab) and GSE109211 (67 HCC patients received sorafenib) were also included in our study (15, 16). All FPKM values were transformed into transcripts per kilobase million (TPM) values. Raw data files of GSE109211 were normalized by lumi (R package). cBioPortal (www.cbioportal.org) was used to download the beta value of DNA methylation status of checkpoint genes from TCGA-LIHC cohort. Gene mutation data (MAF files) of TCGA-LIHC cohort was achieved from TCGA database.

In our previous study, liver tumor tissues from 32 patients in First Affiliated Hospital, School of Medicine, Zhejiang University were collected from November 2013 to July 2014 (GSE138485/PRJNA576155) (17). Only a few of the 32 patients have the available Formalin Fixed Paraffin Embedded (FFPE) specimens. Detailed information of patients is described in **Supplementary Tables S7, S8**.

### Quantification of TME Infiltration

The abundances of immune and stromal cells in TME were quantified by MCP-counter based on cell-type specific transcriptome signatures (10). According to Sylvie’s study (18), a total of 13 TME signatures, which contained 11 stromal and immune cell populations (Lymphoid, B_derived, T_adaptive, Cytotoxic, Monocyte_derived, Myeloid, NK_or_T, Fibroblast, HSCactivated, HSCquiescent, and Myofibroblast) and two functional signatures representing the immune checkpoints (named Checkpoint) and the immunosuppression pathways (named Immunosuppression), were included (**Supplementary Table S1**). In addition, we used the CIBERSORT to validate the immune characterization (11).

### Evaluation of the Immune Score and Stromal Score

The immune score and stromal score of each patient were calculated by ESTIMATE algorithm based on transcriptomic data (19). The R code of ESTIMATE was downloaded from the public source website (https://sourceforge.net/projects/estimateproject).

### Unsupervised Clustering Based on 13 TME Signatures

Based on above 13 signatures, consensus clustering method was used to classify HCC patients into distinct immune subclasses by ConsensusClusterPlus (R package) (20). Detailed settings were as followed: repetitions = 500 times; pItem = 0.8; pFeature = 0.8. The number of the clusters was determined by consensus cumulative distribution function (CDF) curve and the delta area (relative change in CDF area). Because the CDF curve and delta area plot showed that the delta area increased slightly for k = 5 compared to k = 4, we finally selected k = 4 (four immune subclasses) as the best solution (**Supplementary Figure 2**).

### Functional Characterization of Immune Subclass

For pathway analysis of transcriptomic data among immune subclasses, we performed Gene Set Variation Analysis (GSVA) analysis with the “GSVA” R package (21). The gene sets of “c2.cp.kegg.v7.1.symbols”, “c2.cp.bp.v7.1.symbols”, “c2.cp.biocarta.v7.1.symbols”, and “c2.cp.pid.v7.1.symbols” were downloaded from Molecular Signatures Database (MSigDB). Adjusted ANOVA model q value <0.05 was considered as statistically significance.

### Classifier Model Construction and Validation

Based on the 13 TME signatures, we developed a classifier model using Support Vector Machine, as implemented in python package “scikit-learn” (version 0.21.3). The TCGA-LIHC cohort was used as discovery phase, as well as the CHCC-HBV and GSE14520 cohort were validation phases. Detailed information for model construction is described in Supplementary Methods. The efficiency of the classifier model was evaluated by receiver operating characteristic (ROC) curve and the area under the curve (AUC).

### Statistical Analysis

Continuous variables conforming to normal distribution were compared with Student t test, otherwise the Wilcoxon rank sum test was used. One-way ANOVA models and Kruskal-Wallis tests were used for multigroup comparison. The association between immune subclasses and the clinical parameters were evaluated by chi-squared test or Fisher-exact test. Overall survival (OS) curves were calculated according to the Kaplan-Meier method (R package survival) and differences between curves were assessed using the log-rank test. Statistical analyses were performed on R 3.6.2 software and SPSS V26.0 for Windows.

## Results

### Identified Four Immune Subclasses Based on TME of HCC

**Figure 1:**
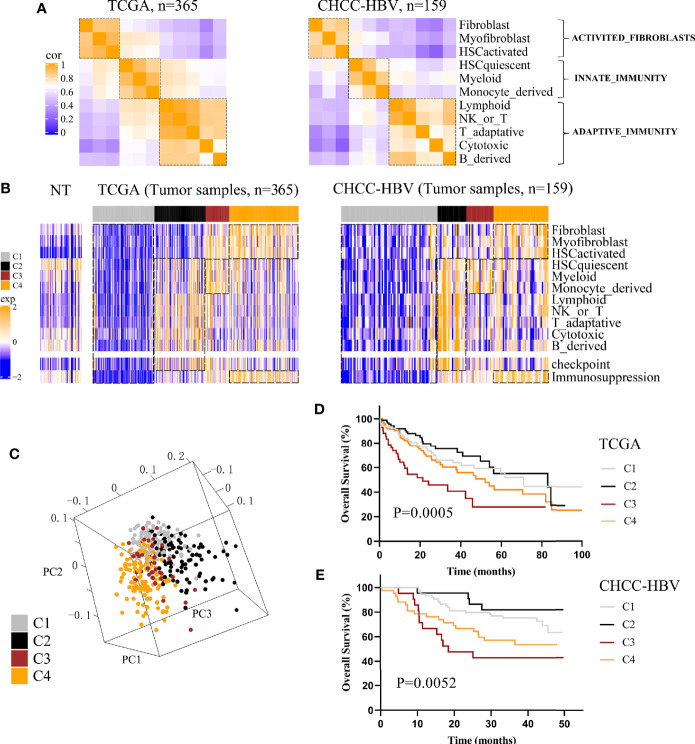
Classification based on tumor microenvironment stratifies HCCs into four subclasses. **(A)** Correlation heatmap of 11 TME cell signatures in two data sets. Color scale: Spearman correlation coefficient from 0 (blue) to 1 (orange). **(B)** Consensus clustering analysis of two data sets revealed four HCC subclasses based on 13 TME signatures. Color scale: Z score from -2 (blue) to +2 (orange). **(C)** Principal-component analysis based on 13 TME signatures separated different subclasses in TCGA cohort. Kaplan-Meier curves of overall survival for TCGA cohort **(D)** and CHCC-HBV cohort **(E)** based on immune subclasses (log-rank test).

Three public HCC data sets with clinical information (TCGA, CHCC-HBV, GSE14520) were enrolled in this study. According to Sylvie’s study (18), a total of 13 TME signatures represented the major infiltrated cell composition and several components of tumor-stroma interaction were included (**Supplementary Table S1**). First, we performed spearman correlation analysis on the 11 cell-type signatures to find the interdependent relationship. **Figure 1A** and **Supplementary Figure 3A** showed that these 11 cell-type signatures were clustered into three distinct clusters (named ACTIVATED_FIBROBLASTS, INNATE_IMMUNITY, and ADAPTIVE_IMMUNITY). In addition, two functional pathways were added, namely, a signature of immune checkpoint related to immune therapy and a signature of genes involved in immunosuppression. Then consensus clustering was performed on the three data sets based on above 13 signatures, and four distinct immune patterns were finally identified, named C1 to C4 (**Figure 1B**, **Supplementary Figure 3B**). Subclass C1 showed an immune desert pattern distinguished by low abundances of all the TME signatures. Subclass C2 displayed an immunogenic pattern distinguished by high abundances of both INNATE_IMMUNITY and ADAPTIVE_IMMUNITY, activation of immune checkpoint pathway and low abundances of ACTIVATED_FIBROBLASTS. Subclass C3 showed an innate immune pattern distinguished by moderate to high abundances of INNATE_IMMUNITY and immunosuppression pathway, rather low abundances of ADAPTIVE_IMMUNITY. Subclass C4 displayed a mesenchymal pattern which was characterized by high abundances of ACTIVATED_FIBROBLASTS and immunosuppression pathway. Principal component analysis (PCA) also showed a significant spatial separation among these four subclasses in TCGA cohort (**Figure 1C**).

Next, we investigated the association between clinical outcomes and immune subclass. In all cohorts, significant differences of prognosis were existed among four immune subclasses, indicating that they could be clinically relevant subclasses (TCGA cohort: log-rank test P = 0.0005; CHCC-HBV cohort: log-rank test P = 0.0052; GSE14520 cohort: log-rank test P = 0.0073) (**Figures 1D, E**, **Supplementary Figure 3C**). In TCGA cohort, C2 Immunogenic subclass showed the longest median survival time (MST) (MST = 82.8 months), followed by C1 Immune desert subclass (MST = 71.0 months), thirdly C4 Mesenchymal subclass (MST = 52.0 months), lastly C3 Innate immune subclass (MST = 21.3 months). Similar results were found in other two data sets. In summary, subclass C2 showed a survival advantage with respect to the other subclasses.

### Immune Functional Characteristics of the Immune Subclasses

To refine the immune characterization, we performed both ESTIMATE and CIBERSORT to calculate the Immune/Stromal scores and the proportion of 22 tumor infiltrating immune cells. C1 Immune desert subclass showed the lower Immune score and Stromal score, while C2 Immunogenic subclass and C4 Mesenchymal subclass showed the highest Immune score and Stromal score, respectively (**Supplementary Figure 4**). CIBERSORT analysis revealed significant difference in 16 out of 22 tumor-infiltrating immune cells, especially an enrichment of CD4^+^ and CD8^+^ T cells in C2, as well as an enrichment of M2 macrophages in C3 (**Supplementary Table S2**). The results of ESTIMATE and CIBERSORT further confirmed the characteristics of the four subclasses defined by MCP-counter.

**Figure 2:**
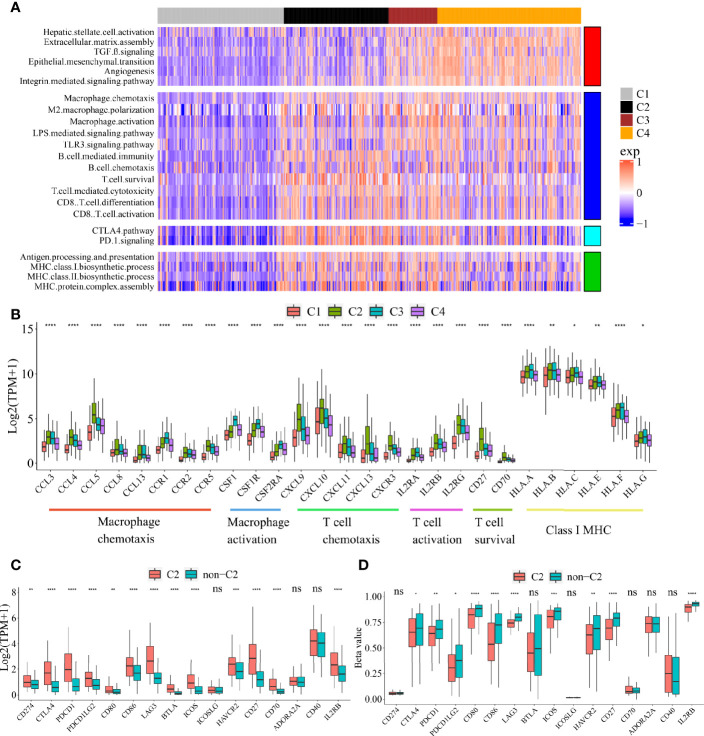
Functional characteristics of four immune subclasses. **(A)** Heatmap of GSVA scores for indicated functional signatures. Color scale: GSVA score from −1 (blue) to +1(red). **(B)** Boxplot plot of the expression levels for selected immune-related pathways. (One-way ANOVA test). Boxplot of the expression **(C)** and methylation **(D)** levels of immune checkpoint-related genes between C2 and non-C2 subclass. (Student’s t test). All P values labels: ns P > 0.05, *P < 0.05, **P < 0.01, ***P < 0.001, ****P < 0.0001). Error bars are presented as the standard deviation (SD).

To explore the functional differences among these four subclasses, we performed GSVA enrichment analysis on TCGA cohort (**Figure 2A**, **Supplementary Table S3**). C1 Immune desert subclass showed a highly attenuation of stromal and immune pathways. C2 Immunogenic subclass showed an enrichment of immune response, such as major histocompatibility complex (MHC) class I and class II biosynthesis, B cell mediated immunity and chemotaxis, T cell cytotoxicity, CD8^+^ T cell activation and differentiation, T cell survival, and immune checkpoint pathway (CTLA4 and PD-1). C3 Innate immune subclass was enriched in macrophage activation, M2 macrophage polarization, TLR3 and LPS signaling. C3 subclass also showed an enrichment of T cell activation and cytotoxicity pathway, but not of T cell survival (different with C2 subclass). These results may account for the lack of adaptive immunity in C3 subclass. C4 subclass was remarkably enriched in activated HSC and stromal pathways such as ECM assembly, EMT, angiogenesis, TGF beta, integrin signaling pathway. The expressions of genes belonging to several immune pathways confirmed the differences among the four subclasses (**Figure 2B**). Markers of macrophage chemotaxis were increased in both C2 and C3, while C3 showed higher expression of macrophage activation. Moreover, the increases in markers of T cell survival, T cell chemotaxis and activation were observed in C2. Markers of all the pathways were low expressed in C1. Specially, compared to the other subclass, C2 subclass showed the both higher expressions and methylation levels of checkpoint-related genes obtained from bulk tumor tissues, which indicated that the overexpression of checkpoints in C2 subclass was potentially triggered by hypomethylation of these genes (**Figures 2C, D****)** (22).

### Mutational Landscape of the Immune Subclasses

**Figure 3:**
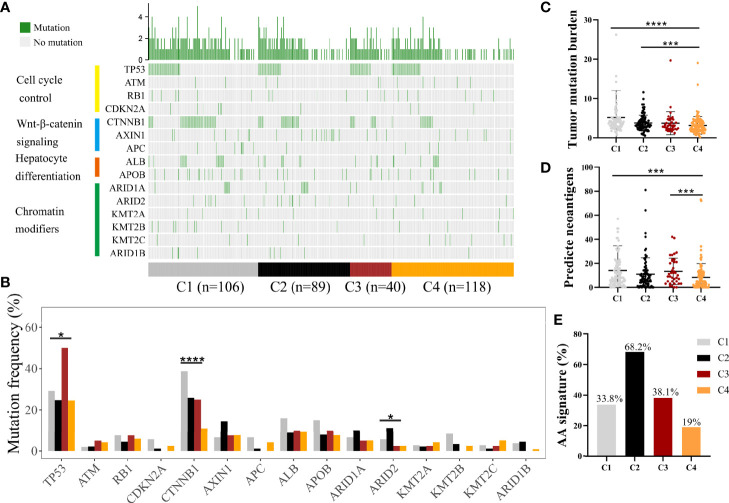
Differences in the mutational landscape among distinct immune subclasses. **(A)** Oncoplot of tumor somatic mutation of genes in P53 pathway, Wnt/beta-catenin pathway, Chromatin modifiers pathway and hepatic differentiation based on TCGA cohort. **(B)** Comparisons of the frequently mutated genes among four immune subclasses based on TCGA cohort. (Fisher’s exact test). Comparison of tumor mutation burden **(C)** and predicted neoantigens **(D)** among four immune subclasses. (Wilcoxon rank sum test). **(E)** The proportion of patients with AA signature among distinct subclasses in CHCC-HBV cohort. *P < 0.05, **P < 0.01, ***P < 0.001, ****P < 0.0001.

Somatic alterations have been proven to be correlated with TME (23). We analyzed somatic mutation data from the whole tumor of several genes with high frequency of mutation and in specific pathways, such as P53-pathway, Wnt-pathway, Chromatin modifiers pathway, and hepatic differentiation from TCGA-LIHC cohort (**Figure 3A**, **Supplementary Table S4**) (24). C1 subclass showed the highest mutation frequency of CTNNB1 (P < 0.0001), while the highest mutation frequency of ARID2 (P = 0.049) was observed in C2 subclass (**Figure 3B**, **Supplementary Table S4**). The highest mutation frequency of TP53 was observed in C3 subclass (P = 0.015). Then, we compared the tumor mutation burden and predicted neoantigens among these four subclasses (**Figures 3C, D****)**. The lowest tumor mutation burden and numbers of predicted neoantigens were detected in C4 subclass. Moreover, in CHCC-HBV cohort, Gao et al. found that signature for aristolochic acids (AA signature) was correlated to tumor mutation burden and response to immunotheropy. In CHCC-HBV cohort, C4 subclass also showed a lower proportion of AA signature than other three subclasses, which indicated a lower benefit from checkpoint blockade therapy (**Figure 3E**).

### Associations Between Immune Subclass and Clinical Characteristic in TCGA and CHCC-HBV Cohort

**Figure 4:**
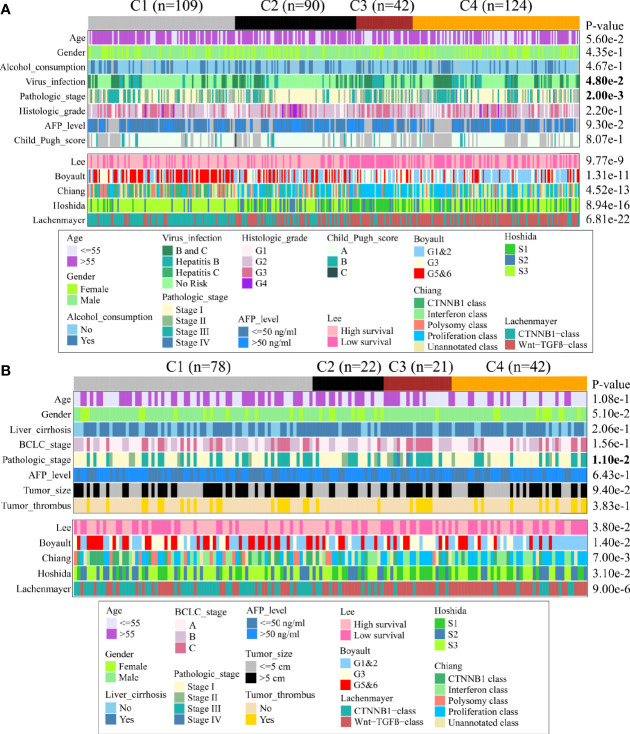
Clinical characteristics of four immune subclasses. Correlation of the immune subclass with clinical characteristics and previously reported HCC classification in TCGA cohort **(A)** and CHCC-HBV cohort **(B)**.

Next, we discovered the associations between clinical characteristics and immune subclass in TCGA and CHCC-HBV cohort (**Figure 4**, **Supplementary Table S5, S6**). The patients from C2 subclass had a higher proportion of pathologic stage I/II (TCGA: P = 0.002, CHCC-HBV: P = 0.011). Patients from C3 subclass showed a higher proportion of HBV infection (P = 0.048). Most of the clinicopathological characteristics did not show significant differences, which suggested that the main factors distinguishing distinct immune subclass were the TME signatures, rather than the above-mentioned clinical features.

Furthermore, we compared our classification with several previous reported classifications based on transcriptomic, including Lee’s classification (High/Low survival), Boyault’s classification (G1 to G6), Chiang’s classification (five classes), Hoshida’s classification (S1 to S3), and Lachenmayer’s classification (CTNNB1 class/Wnt-TGF-beta class) (25–29). In TCGA and CHCC-HBV cohort, C1 subclass was co-clustered with the better-prognosis subclasses (Lee’s High survival, Boyault’s G5&6, Chiang’s CTNNB1 class, Hoshida’s S3, Lachenmayer’s CTNNB1-class). C3 subclass was largely co-clustered with poor-prognosis subclasses (Lee’s Low survival, Boyault’s G3, Chiang’s Proliferation class, Hoshida’s S1, Lachenmayer’s Wnt-TGF-beta class). C2 subclass was linked to both better-prognosis subclass (Lee’s High survival, Hoshida’s S3) and poor-prognosis subclasses (Boyault’s G1&G2, Chiang’s Proliferation, Wnt-TGF beta class). C4 subclass was co-clustered with Lee’s Low survival, Boyault’s G1&G2, Chiang’s Proliferation and Interferon class, Hoshida’s S3, Lachenmayer’s Wnt-TGF-beta class.

### Construction and Validation of a Classifier Based on TME

**Figure 5:**
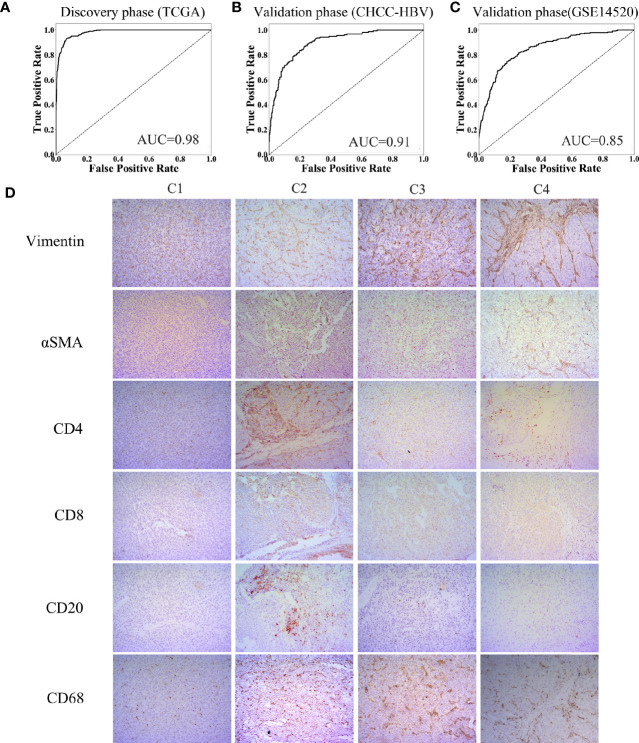
Construction of support vector machine model and performance validation. ROC curves for classifiers designed to predict the immune subclass for TCGA **(A)**, CHCC-HBV **(B)**, and GSE14520 **(C)**. **(D)** Representative immunohistochemical pictures of HCC samples belonging to each subclass (100X) (Zhejiang cohort).

Characteristics of the clinical traits and biological behaviors among four immune subclasses supported our classification. To apply this classification on clinical use, we developed a Support Vector Machine model to classify HCC patients into above four immune subclasses. The input was the 13 TME signatures of each case from the above data sets, and the output was the immune subclass of each case calculated by this model. The ROC curve represents the accuracy between the subclass clustered in **Figure 1B** and **Supplementary Figure 3B** and the subclass predicted by this SVM model. As **Figures 5A–C** showed, these 13 TME signatures revealed a great classification performance in both discovery phase (TCGA cohort: AUC = 0.98) and validation phases (CHCC-HBV cohort: AUC = 0.91; GSE14520 cohort: AUC = 0.85). Furthermore, we applied this classifier model on 32 HBV-related HCC patients (Zhejiang cohort) to divide these patients into four subclasses. We selected a random sample from each subclass to perform immunohistochemical staining for verifying the accuracy of our classifier model (**Figure 5D**, **Supplementary Figure 5**). Several markers of immune and stromal cells were selected, specifically, CD4 and CD8 for T-lymphocytes, CD20 for B-lymphocytes, CD68 for macrophages, αSMA for fibroblastic cells and Vimentin for mesenchymal cells. These markers varied markedly among four subclasses. The expression of Vimentin and αSMA was low in the patient classified into subclass C1, moderate in subclass C2 and C3, high in subclass C4. The patient classified into subclass C2 was characterized by the massive infiltration CD4^+^, CD8^+^ and CD20^+^ lymphocytes. Innate immune cells (macrophages) were also observed in subclass C2. Subclass C3 displayed a high infiltration of macrophages. Subclass C4 contained a low density of macrophages and CD4^+^ T cells. Thus, based on immunohistochemistry, the phenotypic features of HCC tumors were consistent with the classification of our SVM model. The results of immunohistochemistry not only partially proved the accuracy of our model, but also supported the rationality of the classification of four immune-related subclasses.

### Different Sensitivity to Personalized Treatment Among Four Immune Subclasses

**Figure 6:**
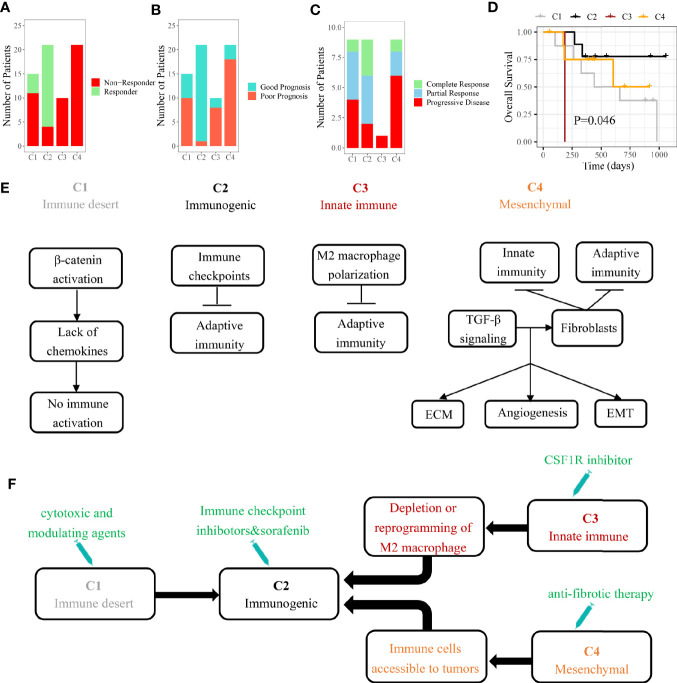
The role of immune subclass in personalized treatment and schematic summary of each immune subclass. **(A)** The number of patients with response to sorafenib. **(B)** The number of patients belonged to good or poor prognosis subgroup. **(C)** The number of patients with response to anti-PD-1 therapy. **(D)** Kaplan-Meier curves of overall survival for GSE78220 cohort (log-rank test, P = 0.046). **(E)** The mechanisms of immune escape for each immune subclass. **(F)** Potential therapeutic strategies for each immune subclass.

In HCC patients with Child-Pugh Class A or B, the multi-kinase inhibitor sorafenib has become the first-line systemic therapy (30). However, there were still no effective clinical characteristics to predict the response to sorafenib so far. Several recent studies suggested that sorafenib may exert the anti-tumor effect by regulating the TME of HCC (31, 32). By our SVM classifier, 67 patients from GSE109211 were divided into four subclasses to explore the associations between the immune subclass and the response to sorafenib. We found that 81% cases of C2 subclass showed a significant response to sorafenib, indicating that patients from Immunogenic subclass were more likely to benefit from sorafenib treatment (**Figure 6A**). As Pinyol and colleagues divided these 67 patients into Good/Poor Prognosis subgroups, C2 subclass was also coclustered with the Good Prognosis subgroup (95%) (**Figure 6B**).

In recent years, some immunotherapies like PD-1 blockade have achieved success in HCC (33). Different immune cell infiltrations and expressions of checkpoint-related genes suggested that four immune subclasses could have the distinct response to immunotherapy (34). We tried to apply our model on another pembrolizumab-treated cohort (GSE78220). The highest response rate (77.8%) to pembrolizumab was observed in patients belonging to C2 subclass with the best outcome (**Figures 6C, D****)**. The results indicated that HCC patients belonging to immunogenic subclass may benefit from anti-PD-1 therapy inferentially.

## Discussion

Although there is a strong heterogeneity in the tumor immune microenvironment of each HCC patient, a clinical benefit could be made from classifying a patient into a specific immune subclass. After analyzing the landscapes of transcriptome, methylation, somatic mutation, and clinical characteristics, we found that these four subclasses may correspond to different mechanisms of immune escape (**Figure 6E**). Immune desert subclass (C1) is characterized by immune ignorance and a lack of priming T cell, corresponding to immune-desert phenotype. The activation of the β-catenin caused by CTNNB1 mutation might account for the low immune infiltration represented in C1 (35). Immunogenic subclass (C2) is characterized by a massive immune cell (CD4^+^ T cell, CD8^+^ T cell, B cell, and macrophage) infiltration in tumor corresponding to immune-inflamed phenotype. Negative regulators of the immune response (PD-L1, CTLA4, etc.) might be involved in counteraction of anti-tumor immune response (36). A mount of patients belonging to C2 subclass showed a low pathological stage (TNM stage). In line with our study, several studies demonstrated that the tumors in low pathologic stage usually infiltrated with numerous immune cells. The patients belonging to C2 subclass showed the highest mutation frequency of ARID2 which was related to the efficacy of checkpoint blockade immunotherapy in clear cell renal cell carcinoma (37). Innate immune subclass (C3) is characterized by the activation of M2 macrophages (related to innate immunity). M2 macrophage, which exerts the anti-inflammatory and immunosuppressive effects, might promote the immune escape represented in C3 subclass through inhibiting the infiltration of adaptive immune cells (38). Mesenchymal subclass (C4) shows a large number of activated fibroblasts (including HSC and myofibroblast) which influence EMT and the sensitivity of drug treatment through synthesizing growth factors, chemokines and adhesion molecules (39).

Kaplan-Meier analysis based on 766 participants showed that significant differences in overall survival were discovered to exist among our four immune subclasses. Immunogenic subclass (C2) represented the best clinical outcome, while innate immune subclass (C3) have the worst. This suggested that the different TME continuously and chronically affects the progression of HCC, which is ultimately reflected in the different clinical outcome. Based on the RNA-seq data from bulk tumor tissues, our convenient classification dividing the patients into four subclasses may infer the prognosis. What is more, the conversions of the immune subclasses by external interventions may benefit the long-term clinical results and outcomes of HCC patients.

Additionally, we established an SVM model based on the 13 TME signatures (11 immune-related cells and 2 immune-related pathways) and confirmed its predictive value (CHCC-HBV cohort: AUC = 0.91; GSE14520 cohort: AUC = 0.85). The input (13 TME signatures) of the SVM model were calculated by MCP-counter based on transcriptomic data, while giving a specific output (which immune subclass). Thanks to the wide applications of RNA-seq, our workflow only required frozen/fresh tissue samples (< 100 mg), as well the model constructed on python was convenient and efficient. The immunohistochemistry from each subclass proved not only the rationality of the TME classification but also the accuracy of the SVM model (**Figure 5D**). Accordingly, this suggested that for any HCC patient undergoing liver biopsy or liver resection, our SVM model can be used to infer the prognosis and guide the follow-up treatment.

HCC patients may benefit from identifying immune subclass which may guide personalized treatment strategies (**Figure 6F**). In our study, we found that the patients belonging to C2 subclass might be more suitable for sorafenib and anti-PD-1 therapy. According to the reported study, the patients belonging to C3 subclass could be treated with colony-stimulating factor-1 inhibitor which improved the efficacy of immunotherapy through inhibiting the intertumoral accumulation of M2 macrophages (40, 41). For the abundant fibrous stroma observed in C4 subclass, anti-fibrosis drugs (like NOX4 inhibitor) suppressed the activation of cancer-associated fibroblasts and promoted the infiltration of CD8^+^ T cells, ultimately improving the efficacy of immunotherapy (42). As for C1 subclass, the application of cytotoxic and modulating agents which can convert cold tumors to inflamed tumors was a potential strategy (43).

Our workflow is limited by the HCC patients obtained specimens for the first time, as well as the influences of confounding variables such as HBV/HCV infection, alcoholic fatty liver, non-alcoholic fatty liver, and cirrhosis were not considered. We will improve them in the future work.

In conclusion, our study dementated a new landscape for the composition of HCC tumor microenvironment. We identified four immune subclasses with distinct mechanisms of immune escape. The patients from distinct subclasses showed a significant difference in clinical prognosis and response to personalized treatment. Based on transcriptome data, our workflow might help to predict the clinical outcome and to find appropriate treatment strategies for HCC patients.

## Data Availability Statement

The data sets presented in this study can be found in online repositories. The names of the repository/repositories and accession number(s) can be found in the article/**Supplementary Material**.

## Ethics Statement

The studies involving human participants were reviewed and approved by the Clinical Research Ethics Committee of the First Affiliated Hospital College of Medicine, Zhejiang University (2014-334). The patients/participants provided written informed consent to participate in this study. Written informed consent was obtained from the individuals for the publication of any potentially identifiable images or data included in this article.

## Author Contributions

Study concept and design: SZ, LZ, and XG. Analysis and interpretation of data: XG. Technical and material support: SY, YW, and CP. Drafting of the manuscript: SZ, LZ, and HH. All authors contributed to the article and approved the submitted version.

## Funding

This study was supported by Zhejiang Provincial Public Welfare Technology Research Program (LGF18C100001). This study was also supported by Innovative Research Groups of National Natural Science Foundation of China (no. 81721091), National S&T Major Project of China (no. 2017ZX100203205) and Research Unit Project of Chinese Academy of Medical Sciences (2019-I2M-5-030). This study was also supported by Zhejiang International Science and Technology Cooperation Project (no. 2016C04003).

## Conflict of Interest

The authors declare that the research was conducted in the absence of any commercial or financial relationships that could be construed as a potential conflict of interest.
