# Supplementary material for: Tumor Immune Microenvironment Characterization in Hepatocellular Carcinoma Identifies Four Prognostic and Immunotherapeutically Relevant Subclasses
Source: Front Oncol. 2021 Feb 19;10:610513. doi: 10.3389/fonc.2020.610513 (PMC7933665; doi:10.3389/fonc.2020.610513)
Supplement: Supplementary file 1 [file DataSheet_1.pdf]

Supplementary figure S1

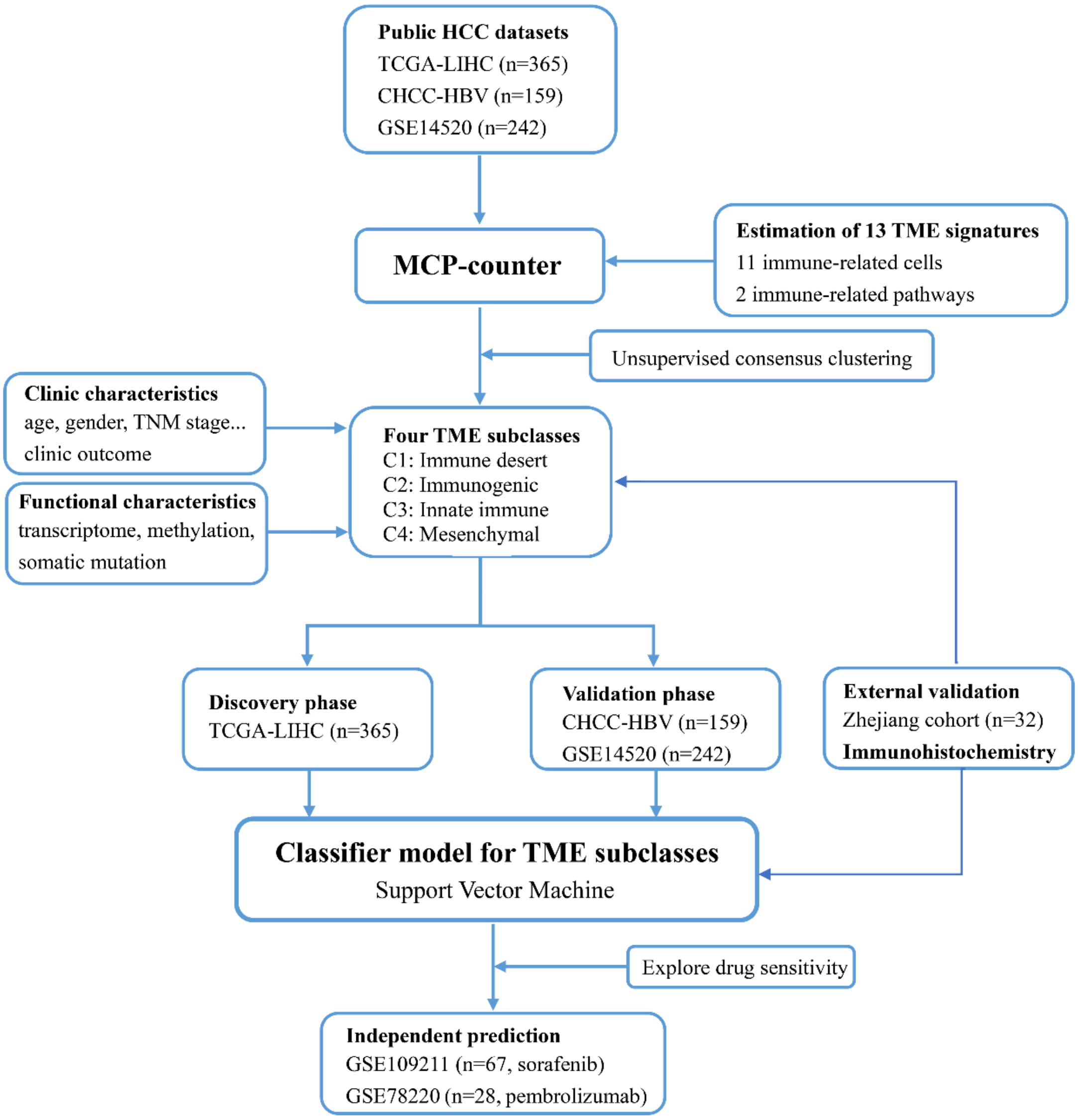

Figure S1. Study design and flow diagram. A total of 766 patients from TCGA-LIHC, CHCC-HBV and GSE14520 cohorts were included in our study. Tumor infiltrating cells and functional signatuers were quantified by MCP-counter. Four immune subclasses were identified through unsupervised clustering. After analyzing the characteristics of functional and clinical characteristics, a Support Vector Machine (SVM) model of 365 patients (discovery phase) and 401 patients (validation phase) was built. Immunohistochemical staining of the samples in Zhejiang cohort was used as external validation. Then the SVM model was applied on another two independent cohorts of patients who received sorafenib/pembrolizumab treatment to explore the drug sensitivity.

supplementary figure S2

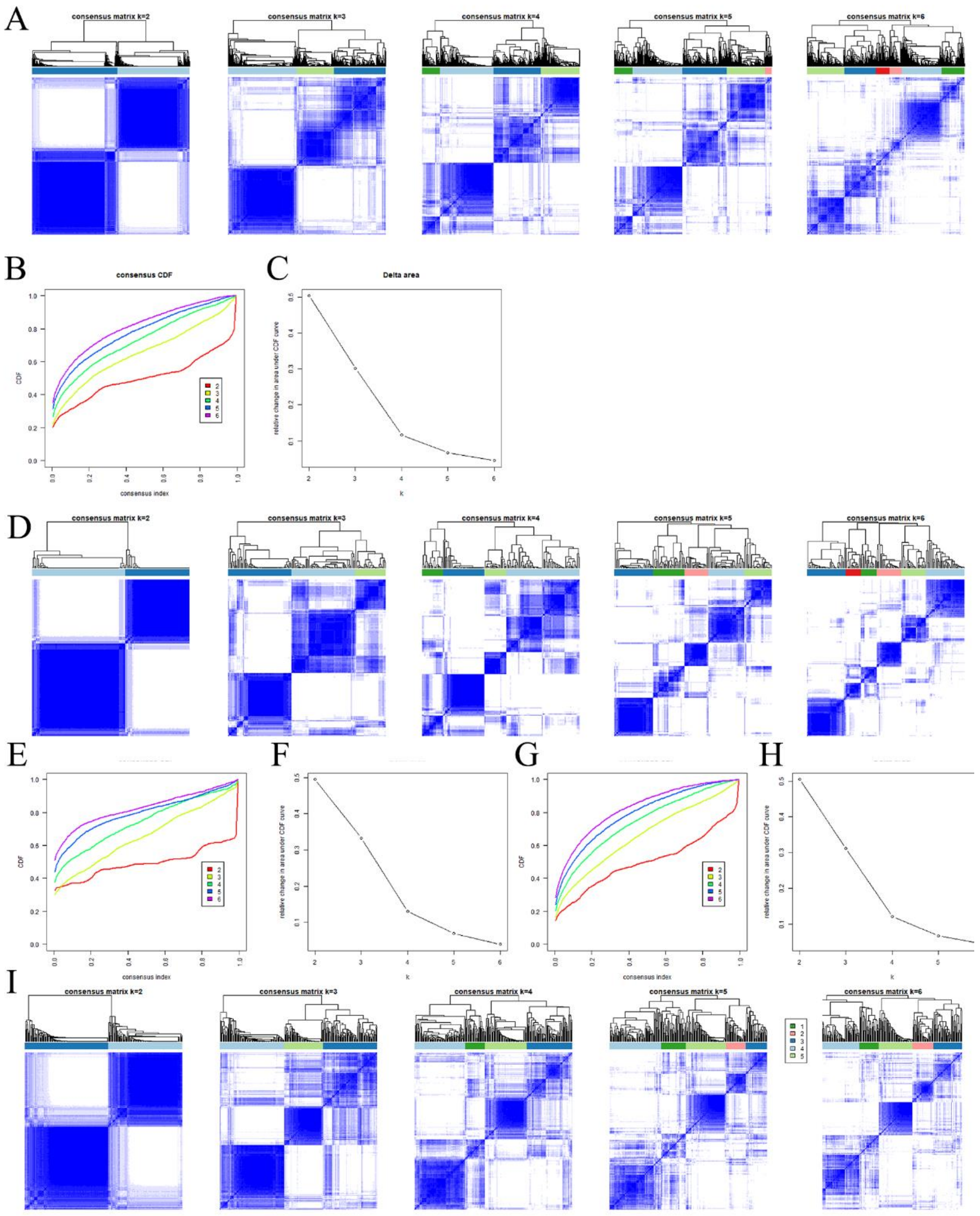

Figure S2. Consensus clustering for 13 TME signatures in three public datasets. The consensus matrices (A), consensus CDF (B) and delta area plots (C) of the TCGA cohort for k=2-6. The consensus matrices (D),consensus CDF (E) and delta area plots (F) of the CHCC-HBV cohort for k=2-6. The consensus CDF (G), delta area plots (H) and consensus matrices (I) of the GSE14520 cohort for k=2-6.

supplementary figure S3

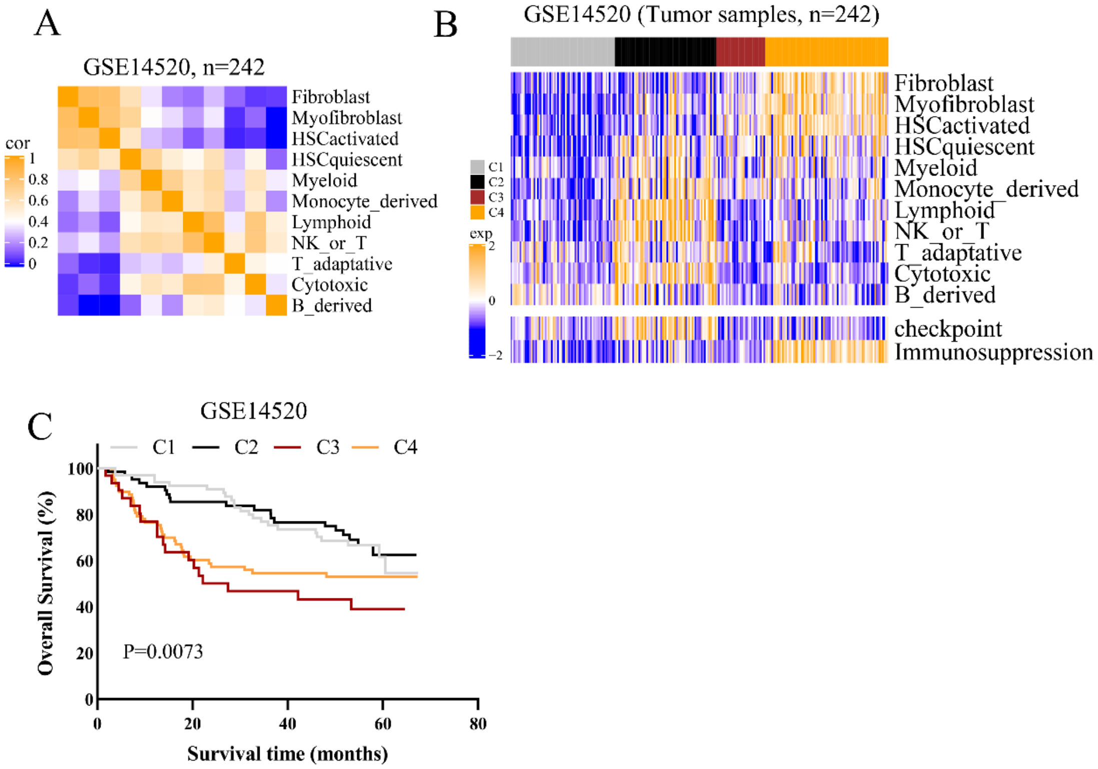

Figure S3. Classification based on tumor microenvironment stratifies HCCs into four immune subclasses in GSE14520 cohort. (A) Correlation heatmap of 11 signatures of immune-related cell populations in two datasets. Color scale: Spearman correlation coefficient from 0 (blue) to 1 (orange). (B) Consensus clustering analysis of two datasets revealed four HCC subclasses based on 13 TME signatures. Color scale: Z score from -2(blue) to +2(orange). (C) Kaplan-Meier curves of overall survival for GSE14520 cohort based on immune subclasses (log-rank test).

supplementary figure S4

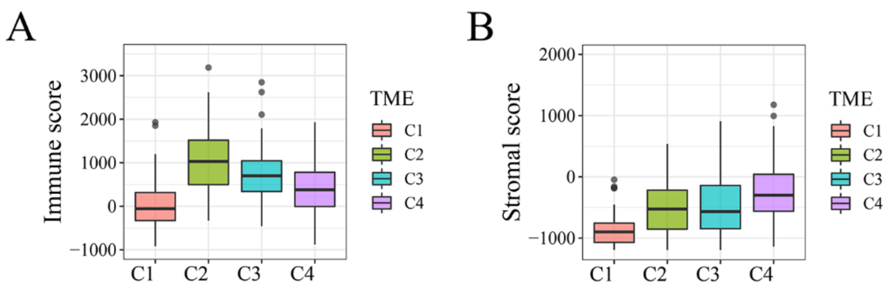

Figure S4. Boxplot of immune score (A) and stromal score (B) from ESTIMATE of four subclasses in TCGA cohort. Error bars are presented as the standard deviation (SD).

supplementary figure S5

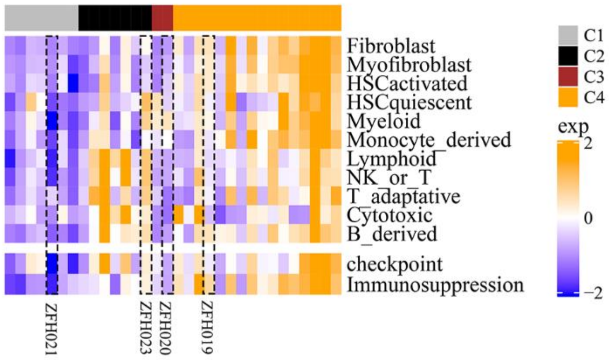

Figure S5. Heatmap of 32 HCC patients from Zhejiang cohort predicted by SVM model. For each subclass, we selected a specific sample to perform immunohistochemical staining. Color scale: Z score from -2(blue) to +2(orange)
