# Supplementary material for: Tumor Immune Microenvironment Characterization in Hepatocellular Carcinoma Identifies Four Prognostic and Immunotherapeutically Relevant Subclasses
Source: Front Oncol. 2021 Feb 19;10:610513. doi: 10.3389/fonc.2020.610513 (PMC7933665; doi:10.3389/fonc.2020.610513)
Supplement: Supplementary file 2 [file DataSheet_2.pdf]

Supplementary Methods

CIBERSORT estimation

The gene expression data with standard annotation were uploaded to the CIBERSORT web portal (http://cibersort. stanford.edu/), and the algorithm was run using the LM22 signature and 1000 permutations. Cases with a CIBERSORT output of $p<0.05$, indicating that the inferred fractions of immune cell populations produced by CIBERSORT are accurate, were considered to be eligible for further analysis. For each sample, the final CIBERSORT output estimates were normalized to sum up to one and thus can be interpreted directly as cell fractions for comparison across different immune cell types and datasets.

Comparison of the our immune subclasses with previous HCC classification

Five reported HCC gene expression signatures, including Lee's classification (High/Low survival), Boyault's classification (G1 to G6), Chiang's classification (five classes), Hoshida's classification (S1 to S3), Lachenmayer's classification (CTNNB1 class/Wnt-TGF-beta class) were collected for comparison. For each subtyping procedure, samples underwent consensus clustering based on each classifiers genes, followed by semi-automatic subtype assignment based on gene expression patterns.

Immunohistochemistry

Formalin-fixed, paraffin-embedded liver tumor sections (3μm) were de-waxed in xylene and rehydrated through graded alcohols. After antigen retrieval, tissue sections were incubated with 0.3% H_2O_2 for 10 min to block endogenous peroxidase followed by incubation with the following rabbit monoclonal primary antibodies: CD4 (abcam, ab133616, 1:250), CD8 (abcam, ab93278, 1:250), CD20 (abcam, ab78237, 1:100), CD68 (abcam, ab213363, 1:500), αSMA (ABclonal, A1011, 1:100) and Vimentin (abcam, ab92547, 1:250) overnight at 4°C. Slides were incubated for 30 min at 37°C with secondary antibody (PV-8000, ZSGB-BIO). HRP activity was detected using DAB+ Substrate Chromogen System (ZLI-9018, ZSGB-BIO). The sections were photographed by microscopy (Zeiss, Germany).

The detailed scripts of SVM model construction for subclass classification (python 3.7.0)

```
import numpy as np
import matplotlib.pyplot as plt
from sklearn import svm, datasets
from sklearn.metrics import roc_curve, auc
```

```
from sklearn.model_selection import train_test_split
from sklearn.preprocessing import label_binarize
import pandas as pd

#loading data of discovery phase and validation phase
df_train = pd.read_csv('#discovery phase data file')
## train
X = df_train.loc[:,'Fibroblast':'Immunosuppression']    # 13 TME signatures
y = df_train.loc[:,'label']             #four subclasses
y = label_binarize(y, classes=[0,1,2,3])
n_classes = y.shape[1]
X_train=X
y_train=y
df_train = pd.read_csv('#validation data file')
X = df_train.loc[:,'Fibroblast':'Immunosuppression']      # 13 TME signatures
y = df_train.loc[:,'label']          #four subclasses
y = label_binarize(y, classes=[0,1,2,3])
X_test=X
y_test=y


random_state = np.random.RandomState(1)          # random number generation
n_samples, n_features = X_train.shape

# train and test of svm
classifier = OneVsRestClassifier(svm.SVC(kernel='linear', probability=True,C=5,
                                 random_state=random_state))
y_score = classifier.fit(X_train, y_train).decision_function(X_test)

# compute ROC curve and area the curve
fpr, tpr, _ = roc_curve(y_test.ravel(), y_score.ravel())        #false positive, true positive
roc_auc = auc(fpr, tpr)
plt.figure()
plt.plot(fpr, tpr,
         label='micro-average ROC curve (area = {0:0.2f})'
               ''.format(roc_auc),
         color='black',    linewidth=4)
plt.plot([0, 1], [0, 1], 'k--', lw=lw)
```
