# Supplementary material for: Tumor Immune Microenvironment Characterization in Hepatocellular Carcinoma Identifies Four Prognostic and Immunotherapeutically Relevant Subclasses
Source: Front Oncol. 2021 Feb 19;10:610513. doi: 10.3389/fonc.2020.610513 (PMC7933665; doi:10.3389/fonc.2020.610513)
Supplement: Supplementary file 3 [file DataSheet_3.pdf]

Operation informed consent

(Translated from Chinese)

1. This is a notice about surgery/operation. The purpose is to tell you about the surgery, diagnosis or treatment related to your doctor's advice. Please read carefully and ask any questions related to this operation to decide whether you agree to the operation or operation.
2. Due to known or unknown reasons, any surgery or operation is potential to: fail to achieve the expected results;lead to complications, injuries and even death. You have the right to know the nature and purpose of the surgery/operation, the risks that exist, the expected results or the impact on body. Except in the event of a life-threatening emergency, without giving your knowledge and obtaining written consent from you，The doctor can't perform surgery/operation on you.You have the right to accept or refuse surgery/operation at any time prior to surgery/operation
3. Your primary doctor:__________ Your Economic doctor:________

   Preoperative diagnosis: ______________________________________

   Proposed surgical/operation name：____

   ____

   Need partial surgery：□Yes □No
4. The doctor will explain：

   4.1 The nature, purpose, and expected outcome of surgery/operation：

   ____

   4.2 any possible associated discomfort, complications or risks：

   a Accidents and dangers that may occur during surgery：

   1. Anesthesia accident 2. Uncontrollable major bleeding

   3. Drug allergy 4. Death or irreparable brain death

   5. Heartbeat and respiratory arrest

   6. interruption of the surgical procedure or changes to the surgical plan

   7. Unavoidable damage to adjacent organs, blood vessels, nerves, etc., which may lead to patient disability or functional damage

   8.Others:____

   b Accidents and complications that may occur during surgery

   1. Postoperative bleeding 2. Local or systemic infection

   3. Incision splitting 4. Organ failure

5. Water and electrolyte balance disorder

6. Postoperative airway obstruction   7. Breathing, cardiac arrest

8. deterioration of the original disease

9. Postoperative pathology report does not match the results of intraoperative frozen pathology

10. Surgery again

4.3 Precautions to be taken in response to the above situation

Based on the various complications that may occur during and after surgery, we will take the following precautions to maximize patient safety and smooth the treatment process according to modern medical regulations. Specific measures are:

1) Preoperative:1.Preoperative: Carefully evaluate the patient and select the appropriate surgical procedure. 2. Improve the necessary preoperative examinations, such as clotting time, liver function, preoperative immunization, electrocardiogram, chest X-ray, etc.

3. Targeted treatment based on underlying diseases.

2) Intraoperative: 1.Closely monitor changes in vital signs, keep vital signs stable, prepare various first-aid equipment, and handle various situations during surgery; 2.Careful operation, gentle and accurate movements. 3.Strictly hemostasis.

3) Postoperative: Close monitoring of vital signs and changes in surgical site, and targeted use of anti-inflammatory, hemostatic drugs and other symptomatic treatments

4) If necessary, consult the relevant department for consultation

4.4 Alternative treatment options:___________________________________

The treatment plan you choose:_______________________________

5. For implants, select the type you want:________________________________

6.Your chief surgeon:____________   assistant:___________________

7. The proposed surgery/operation will be carried out according to your authorization and consent. If there is an emergency or unexpected situation, the doctor will contact the family in time. According to the situation, the doctor will take any necessary measures according to professional judgment.

8. If your doctor believes that you need blood transfusions or blood products during surgery/operation, he/she will tell you about the risks of blood transfusions or blood products, including hepatitis, HIV, etc. due to blood transfusions or blood products.

Therefore, you have the right to consent or refuse to accept blood transfusions. Any questions you have about blood transfusions can be discussed in a timely manner with your doctor.

9. **Your signature indicates that you have authorized the pathologist to perform the necessary medical treatment and scientific research on the relevant tissues and organs removed during the surgery/operation.**
10. **Your signature indicates that you agree that the learner will observe during the inspection process and agree to take a photo that does not indicate your identity (possibly to publish it) for medical and teaching purposes**.
11. In order to ensure an accurate understanding of the above, the doctor will explain the above before you read the informed consent and make decisions. If you have any other questions, I hope you will tell your doctor in time.

Your signature below means:

1. You have read and understood and agreed to the content described above.
2. Your doctor has fully explained the above situation.
3. You have obtained information about the operation. 4. You authorize the doctor to perform the above surgery/operating for you.

doctor signature___________ signature date __________

patient signatire____________ signature date __________

□ patient □ spouse □ offspring □ parent □ Other relatives

□ friends □ others
